# Medical Merchandising and Legal Procedure in Late Sixteenth-Century Spain: The Case of Petroleum as Imported Medicine

**DOI:** 10.1093/shm/hkz032

**Published:** 2019-04-30

**Authors:** Ted Lars Lennart Bergman

**Keywords:** Spain, Italy, petroleum, medicine, lawsuits

## Abstract

This article examines the historical context and particular case of Italian merchant Guido Mondones (also named as ‘Modones’) who sold petroleum as medicine in Spain in the late sixteenth century. The first two-thirds of the article uses printed sources as a way to demonstrate that this merchant was likely not disadvantaged by being a foreigner and itinerant. Nor would he have been considered a suspect for selling oil with healing properties, as it was a fully accepted practice, including among university-trained professionals. All the materials for Mondones’s particular case are archival and contained within a lawsuit from Valladolid that contains a wealth of information about the merchant’s relationship with legal and medical authorities. Through these sources, we learn that he managed to be financially successful by navigating Spain’s particular medical–legal landscape at the time and skillfully defending himself against accusations of tax evasion and selling false medicine.

##  

In 2014, the editors of *Medical Cultures of the Early Modern Spanish Empire* warned that:

Academic medicine was not the predominant or most pervasive medical culture in the early modern Spanish empire. In light of this, we believe it to be necessary to interrogate the way historians have generally constructed medical hegemonies and hierarchies in order to make room for conceptualizations of order that may be non-hegemonic, temporary, fluid, and even unstable. Reducing early modern Spanish medical history to an oscillating binary (academic medicine falls while extra-academic medicine rises) is not the answer. Instead, it is necessary to have a broad understanding of the medical cultures operative in specific locales at particular times …^1^

This persistent binarism, whether it oscillates or not, is reinforced by an imbalance between an abundance of printed academic medical sources, on one hand, and less accessible examples of extra-academic practice, on the other. It is therefore useful to discover and elucidate real cases that are specifically ‘non-hegemonic, temporary, fluid and even unstable’, including cases in which the acceptance of extra-academic practice by contemporary academics functioned to dissolve or elide certain hierarchies. The term ‘extra-academic medicine’ is a calque of the Spanish phrase ‘medicina extraacadémica’, an imperfect term meant to include those whose practice was not based on traditionally academic galenic principles. Extra-academics were not medical professionals, not physicians, surgeons or apothecaries, since all of those underwent a regulated and controlled form of training. In 2002, María Luz López Terrada noted that ‘medical historiography has paid scant attention to these other medicines’.^2^ This present article aims to contribute to a thriving scholarship that has grown over the last two decades and which continually seeks finer distinctions between the many categories of extra-academics. At the same time, from the start, it is not always easy to disentangle one camp (‘academic’) from another (‘extra’), especially as the absolute distinctions between the two have been ‘deconstructed’ over time.^3^ Sabrina Minuzzi nicely lays out two models employed by other scholars for making more subtle distinctions, either ‘core-versus-penumbra’ or ‘medical marketplace’, while she also explains how each model requires its own refinement.^4^ Minuzzi’s article proves that while the permeability between academic and extra-academic practice can cause some confusion, both then and now, studying early modern attempts at clarification can offer great insight into the diverse legal, economic and social systems in operation. Most recently, María Luz López Terrada and Carolin Schmitz have done this in the context of those who ‘acquired their knowledge of cures in an irregular manner [and] were forced to use other means to construct their identity and recognition as medical practitioner, that is to say, obtain a social license for healing’.^5^ This study will add to the richness of recent scholarship by presenting in its second half the case of Guido Mondones, an Italian merchant prosecuted for not paying tax while selling medical petroleum that he had brought over from his home region of Modena. Mondones’s spirited legal defence reveals how he functioned in conjunction with, not in opposition to, the discourses and methods of legal and medical authorities who ultimately supported his endeavours. Starting with historical context and moving towards the specific case study, the article has three goals in mind. The first goal is to show how unstable categories can be paradoxically used to reveal a clear and consistent vision of extra-academic practice. Mondones, who appears to have had no medical training, is named as a ‘merchant’ in legal documents, and we shall use that term here. But he could also be classified as something between a ‘merchant-charlatan’ and ‘pedlar-charlatan’, to use David Gentilcore’s terms, since Mondones was wealthy and produced his own handbill (as would a ‘merchant-charlatan’) but did not concoct his own medicine (as neither would a ‘pedlar-charlatan’).^6^ By confronting this difficult categorisation, we are forced to reckon with the exact details of the merchant’s social, legal and commercial status. In this way, the strict labels of ‘false doctor’, ‘charlatan’ and ‘pedlar’ that populate fictional or anecdotal printed accounts are revealed as inadequate to sum up the variety of experience in extra-academic practice. The second goal of the article is to explain the importance of the legal system in Mondones’s success, how he turned obstacles into advantageous safeguards against legal prosecution. At first glance, lawsuits related to extra-academic practice in the Spanish archives may seem to reinforce stereotypes of deviousness, of charlatans’ false promises; but Mondones’s case reveals how much the law could be on a practitioner’s side, especially when the defendant was assisted by expert medical opinion. The third goal, related to the second, is to tell a possibly exemplary tale of a medical salesman from Italy who, according on the available evidence, could hardly be called a marginalised figure. Based on stereotypical portrayals in the theatre of the time, to be an itinerant merchant and a foreigner in Spain might seem like two strong reasons not to be taken seriously, but Mondones’s case proves otherwise.^7^ I write ‘possibly exemplary tale’ because this article is structured to show that Mondones was likely not alone. The final third of this article uses his specific case to start on the path to reaching the three goals stated earlier. The first two-thirds seek to provide sufficient background to demonstrate that there were several factors in place, such as the legal system, the integration of Italian practitioners in Spain and a tradition of selling simple oil as a remedy, that allowed for Mondones’s success and that this success could be duplicated by others who were often extra-academic in background. Certainly, until we find more cases much like Mondones’s, it is wise to temper some of this zeal for generalisation. This study is laid out to encourage others to seek examples that confirm the need for the research demanded in the quotation used at the start of this article. Lastly, for specialists in the history of medicine in Spain, this study provides one of the few original accounts on the practical use (supply, demand, legal status) of petroleum in Spain that historiography has recently seen.

Because the majority rarely wrote its own history, we must look beyond the written treatises of elite physicians to find it. For many of those who made their living through medicine without an officially regulated training, their story remains to be told. Handwritten legal documents such as lawsuits contain a wealth of information and provide a valuable counterpoint that supplements the study of published sources and can also be used to examine the day-to-day consequences of plying one’s trade far from home.^8^ By studying the medical–legal landscape of late sixteenth-century Spain and examples of well-known Italian practitioners who sought their fortune abroad, it will become clear that the law could create obstacles as much as it could offer protection. A successful merchandiser of medicine in Spain needed to make the system work for him or her. To gain maximum advantage in the marketplace, it was necessary to turn public victories into both promotional material and a storehouse of documents for future legal defence. Written sources on medicinal petroleum and medicinal oils in general provide further evidence of how a merchant for such cures could use medical and legal precedents outside of his or her own experience for self-promotion and protection in a court of law. Before we arrive at Guido Mondones and his ‘oli petroli’, it is useful to provide some historical background and cases from printed sources to show that his story is not that of an outlier but rather of a man who would have felt at home, commercially and legally, while abroad. Selling rock oil in early modern Spain was not the same thing as selling ‘snake oil’ today, and there is no evidence that Mondones was shunned or seen as a fake by professionals. In fact, when he was accused of fakery, it was professional physicians who defended the purity and efficacy of the Italian’s medicinal petroleum. Professional jealousy among medical practitioners in Spain could be felt on all levels, but there were occasions when different actors saw their jobs as belonging to a separate yet complementary sphere of influence, as we shall see in the next section of this study. Mondones must have anticipated antagonism and legal entanglements as he sold his medicine, but he would not have been entirely surprised to find some mutual understanding between merchants and physicians since it also occurred in his home country of Italy.^9^

As María Luz López Terrada and Alvar Martínez Vidal succinctly state:

In the context of the Hispanic Monarchy during the early modern era there was an institution, the Royal Tribunal of the Protomedicato, created by the Catholic Monarchs initially for the Castilian Crown, an institutional framework through which the incipient Modern State tried to control everything related to the exercise and practice of medicine…^10^

Despite its great authority, it was impossible for the Protomedicato to control every aspect of medicine.^11^ Two quotes from dialogues found in Enrique Jorge Enríquez’s *Portrait of the Perfect Physician* (1595) give a contemporary account of how the system was far from perfect:

What I am mocking are the wretches who in the streets, on the corners, and in the herbalists shops shout and claim that they have knowledge from medical authorities and that they know them by heart, so that the common people take them for wise men. They take the patient’s pulse, then they say some half-Latin word, and presenting the patient with his urine they say ‘oppilatio’, giving it dozens of swirls: all of which is against all the laws of medicine [‘regla de la Physica’], so that with these fabrications and tricks they deceive the common people. For the most part, these [practitioners] are a bunch of charlatans, prattlers, and buffoons [‘charlatanes, habladores, juglares’], all of a quality that no perfect physician should have.^12^

These words from the *Licenciado* character in the dialogue are more than typically broad prose satire. They must be seen in the context of some very specific criticism that appears later in the book:

…and [Galen] asks that the Physician consider that … he is not dealing with stones, bricks, sticks, and skins, like other artisans. And for all this I see that the illustrious and noble *protomédicos* can easily give two thousand idiots in this kingdom a license to cure, and to deal in things touching on such an excellent craft. If this is not remedied, why do we dedicate so much study and spend so much money on Philosophy and Medicine?^13^

Enríquez’s complaint is an academic doctor’s personal invective aimed at empirics, but it also sits within a broader and longer lasting debate about competing jurisdictions and medical authority in Spain.^14^ In 1523, the *Cortes* of Valladolid were at the centre of conflicting control over practice. Despite the establishment of the Protomedicato:

…[the *Cortes*] make it known to Your Majesty that the *protomédicos*, for a modest price and out of self interest, give licences to unqualified people with little experience in surgery or medicine, resulting in great harm and damage. Therefore, we ask Your Majesty to order that those physicians and surgeons licenced by the aforementioned *protomédicos* be reexamined by the authorities and aldermen in the place where those physicians and surgeons practice their profession…^15^

This same document also demands that inside a radius of five leagues from the Court, the Protomedicato examine and grant licenses to ‘physicians, surgeons, apothecaries, and barbers’; but the institution is not to meddle with licensing ‘faith healers [*ensalmadores*], nor midwives, nor spice merchants, nor druggists’.^16^ Following the *Cortes’*s request, the law of the land was aimed at separating extra-academic practitioners—whom Enríquez calls ‘wretches’ shouting in the street—from the true professionals, namely surgeons, apothecaries and barbers with their respectively documented qualifications. And yet local authorities were not satisfied to simply regulate the least qualified of practitioners. Thirty-five years later in 1558, the *Cortes* in Valladolid returned with new demands, including a stringent set of requirements and compulsory paperwork for examining physicians. It was imperative that ‘apothecaries, barbers, druggists, and midwives be licensed by the local authorities and aldermen in the cities or towns where they are to practice their profession, and that those carrying out the licensing exam include two knowledgeable and experienced physicians…’.^17^ In this same petition, the local authorities insisted upon their own jurisdiction as much as possible. ‘We petition Your Majesty that henceforth he not provide the said *protomédicos*, and that he order that there be no more: and if one were already provided, that the *protomédico* be suspended or revoked’.^18^ The legislative and jurisdictional wrangling was unending, and within five years, the *protomédico* himself stepped forward to respond to legal overreach at the local level. During the *Cortes* in 1563, he complained that:

…some places, especially the city of Granada, had given licences to people for healing, and not only to practice in their own areas, but outside of them, throughout the kingdom; and this is a most damaging thing that goes against the preeminence of His Majesty; and his *protomédico* asked that regarding [false licences] … a manner be provided to stop the problems that came about, according to what had been explained. The kingdom responded to the *protomédico* that it would deal with this in terms of what was best for the kingdom.^19^

This is the background against which one can imagine thousands—if we are to believe Dr Enríquez—of medical practitioners of all sorts plying their trade. These individuals could either be university-trained physicians, master apothecaries and surgeons with documented training or people occupying a grey area among the ‘charlatans, prattlers and buffoons’ that Dr Enríquez railed against. All practitioners, whose training and knowledge varied greatly, needed more than their medical skills in order to make a living, something Guido Mondones must have known well in his study of the medical–legal landscape. He needed to skillfully navigate this terrain in his quest for required paperwork, not just to make a living but to defend himself against prosecution or harassment.

##  

Some medical practitioners enjoyed widespread fame and a reputation that preceded them, while others were smaller operators who relied more on their wits and salesmanship than a medical degree or royal *bona fides*. To this latter category, we can more safely apply the label ‘charlatan’.^20^ In a 1588 treatise, Francisco Díaz reflects upon the successful legacy of a certain surgeon named Doctor Romano. Díaz laments that few have really benefitted from the example that the good surgeon provided in curing urinary tract infections. Worse than that, those seeking relief were obligated to ‘flee from charlatans, as these only show proficiency in the tongue, and in their boldness. Since they are foreigners and speak in an obscure manner, it seems like they are saying something, but it is really a mess of words [‘de do diere’] …’.^21^ In his moral treatise *La philosophia vulgar* (1568), Juan de Mal Lara glosses a lesson on bluster taken from Aesop’s fables with the story of a confrontation between a recent medical graduate and charlatan selling medicine in the town square. Mal Lara’s anecdote explicitly uses the word ‘charlatán’, and the figure in question defeats his more qualified opponent by undermining the doctor’s confidence and turning the audience against the man with a medical degree. When the opponent cannot respond to the charlatan’s foreign phrases in German and Flemish, which supposedly represent ‘the authorities of the original texts in Arabic and Greek’, the crowd gathers around the vanquished physician and shouts at him to go home and study.^22^

The use of ‘foreign phrases’ reminds us that charlatans in Spain could come from anywhere and even misrepresent their nationality as a means of self-promotion.^23^ And yet, as a place of origin, Italy stands out in the case of non-native charlatans and not only because the word itself is Italian in origin. Written descriptions and documented cases indicate that this particular profession and particular nationality, while by no means synonymous, were heavily associated in the early modern Spanish imagination. An example from the lexicographer Sebastían de Covarrubias demonstrates how the association goes beyond etymology and into the realm of public perception.

‘Charlatan’ is an Italian word … Charlatans are certain people who go about the world: known by another name as *saltaenbanchi*, because in the town squares they stand upon a table of the sort meant for selling wares, and sometimes with a guitar or viola they sing some song. They usually bring with them a *zanne*, which in Spain is like Foolish John [‘Juan Bobo’] who, with a half-mask and a linen garment, dances and has some funny dialogues with his master. After this, when people have come and gathered, the charlatan opens his box and takes out different little bottles of oils, salves, herbs, roots, and stones: there is no sickness that they do not cure.^24^

Cristóbal Suárez de Figueroa, in translating Tommaso Garzoni’s *La piazza universale di tutte le professioni del mondo*, preserves Garzoni’s observation in the text after citing Flavio Biondo that ‘In our times, the number of these charlatans grew to such an extent that they spread like a weed through all the cities of Italy where one finds a great quantity of them’.^25^ Suárez’s version parallels the original brief sketch of how charlatans captivate their audience, but then he diverges from Garzoni’s account.

After a long speech that lasts more than two hours … [the charlatans] propose to sell small balls of soap, conserves for fortifying gums and teeth, grease made from veal or kid, what they call *pomada*, unguent for scabies or for burns, and things like that. They sing the infinite praises [‘Encaraman su virtud todo lo posible’] of these medicines, setting various prices for them, until they name the lowest, at which point people start to throw them money tied to handkerchiefs.^26^

These additional details could easily portray migrant Italian charlatans’ within Spain, perhaps a cohort from those who ‘spread like a weed’ in their home country and sought less competition abroad. It is also possible that the charaltans in Suarez’s descriptions were not always Italian. Some may have simply exploited an air of Italianness that was part of successful salesmanship, much like the charlatan in Mal Lara’s anecdote cited above who used German and Flemish phrases to bolster his authority. Whether or not they assumed an Italian identity or were authentic, it seems like many merchants of medicine in Spain saw no problem with presenting themselves as Italian despite written objections to them being a ‘weed’ that had spread too far.

Not all such figures were at the mercy of others’ descriptions on the printed page. Some let their own voice be heard through printed advertising leaflets, as we shall see in the case of Guido Mondones and others further. Others went far beyond shouting and leafleting in the town square and invested heavily in the printing side of things. As an example, we find Alessandro Quintilio, who started out in Naples and traveled to Portugal and Spain before settling in Madrid, where he published a work on his ‘quintessence-of-gold powder’. This book-length advertisement from 1607, full of testimonials and self-promotion, went through a second edition in 1609 that was subsequently reprinted in 1616.^27^ Quintilio neglected to mention in his first edition that the powder was not his invention, but rather that of Vittorio Algarotti, whose medicine Quintilio distributed and who was himself an outsized promoter of his product.^28^ While Algarotti graduated in medicine from the University of Padua and later joined the College of Physicians in his home city of Verona, Quintilio appears to be nothing more than a salesman and follower.^29^ In this way, he belonged to an extensive tradition of Italian extra-academics who exploited others’ books of secrets.^30^ Quintilio buttressed his qualifications and fortified his pre-emptive defences against accusations of fakery by citing legal challenges from which he had arisen victorious. He proudly stated in his 1616 edition’s introductory summary:

For twenty-two years, Alexandro has administered these powders in the capital [‘la Corte’], always by the grace of Our Lord, always with great success, and has done so for sixteen years with licence from His Majesty’s *protomédicos*. On two different occasions, the Protomedicato’s prosecutors [‘fiscales’] have wished to speak out against the aforementioned powders, after which he was absolved by both, and freed from the motions against him.^31^

Guido Mondones, whose case will be examined extensively at the end of this article, would have relied upon a general acceptance of Italian practitioners in the Iberian Peninsula and not necessarily suffered any setbacks by virtue of his foreign-ness. In any situation in which he was seen as an unwanted interloper, his vigorous defence was unlikely exceptional, considering the example of Quintilio earlier.

The migration of Italian charlatans to the Iberian Peninsula has yet to be exhaustively documented, and there is insufficient space to do so here. At the same time, there is documentary evidence to suggest that this is an avenue of research worth pursuing further. For example, in David Gentilcore’s database of Italian charlatan licences, we find a certain Domenico Manecatto from Pesaro, who in 1594 obtained a license in Siena for selling ‘terra maltese (earth)’ and ‘powder for teeth’. In 1597, a Domenico Menegati from Pesaro was licensed in Venice to sell a ‘secret for burns’.^32^ In 1614, according to the records of the Archivo del Reino de Valencia, there is a ‘Licence given in Valencia, granted by the doctors Jeroni Garcia and Melcior de Villena, royal examiners of the city of Valencia, in favour of the Italian, Domingo Manegati from Pesaro, after he had presented documents accrediting the practice of his profession in other kingdoms and that he had been licenced [‘examinado’].’^33^ As another example, we find the 1606 case of ‘Joseph Balsamo or Jusepe Valsamo … an Italian who, had arrived in Valencia after selling his medicine in various cities on the Peninsula, and who was authorized to sell as an itinerant merchant in the city a medicine called “medicinal of *germania*”, composed and manufactured by him’. In documenting the case, María Luz López Terrada continues to write that the medicine was for ‘“all manner of illness related to cold”’ and ‘the case dealt with an oil, and not some other liquid [‘un licor’] as stated in [Balsamo’s] advertising broadsheet’.^34^ Many of the healing properties listed on the broadsheet overlap with those claimed by Guido Mondones for his ‘oli petroli’ and place him in the category for what Gentilcore would call a ‘merchant-charlatan’. One may debate the applicability of the term, since it will become evident that Mondones did not concoct his own medicine, and instead seemed to be providing a well-documented simple ingredient. And yet, he may have still had to contend with customers or commercial rivals whose biases had been negatively influenced by satires, written anecdotes and treatises vilifying charlatans. As we shall see from legal documents cited in his case further, Mondones did face accusations of selling false medicine, although he did not defend himself in book form like Quintilio.^35^ Instead, he appears to have relied upon the courts and their documents alone, while his case gives us further evidence of how a foreign seller of medicine could be a success and ultimately escape charges of devious charlatanism.

## Petroleum as Medicine: Medical Authority and Precedents to Its Sale in Late Sixteenth-Century Spain

When Mondones came to the Iberian Peninsula to sell his medicine, it would not have been wholly unfamiliar to those who were literate and interested in such substances. The very word ‘petroleum’ has been used in Europe since the twelfth century, and it is in a medical context that we find the earliest written example.^36^ As it continued to be listed in learned books, it also announced itself through more popular and ephemeral printed media. In one of his books on the history of this rock oil, R.J. Forbes dedicates an entire chapter to ‘Advertizing the Medicinal Properties of Petroleum’, basing his study on four promotional broadsheets printed from 1480 to 1550.^37^ The four pamphlets cited by Forbes are printed either in French or German and three of them make important mention of either ‘Montesible’, ‘Sibia’ and ‘Sybia’, referring to Monte Zibio. This mountain near Modena was a well-known source of petroleum and the three Zibio-related pamphlets in question also contain abundant references to ‘ancient medical authorities’.^38^ Guido Mondones, a man from Modena and whose advertising we shall look at below, had the perfect surname for creating an authoritative persona as a merchant selling a substance from that region known for petroleum production. Pamphlets like the ones cited earlier are the predecessors of Mondones’s own advertising and those that followed him and are part of a sort of parallel papertrail of references to petroleum that would have made it a familiar substance to many at the time. In the anecdote from Mal Lara mentioned earlier, the ‘authorities’ cited by the charlatan in the public square were pure invention; but for a seller of petroleum, it would be much more valuable to cite published experts in support of his claims. Naming real names would earn the confidence of consumers and pre-emptively refute accusations of false medicine or false practice that local authorities might level against him. In the specific case of petroleum, law-enforcement officials and medical professionals had recourse to a number of books printed in Spanish that referenced the substance, often called ‘naphtha’. Doctor Andrés de Laguna, physician to the Emperor Charles V, Pope Julius III and King Philip II, wrote about naphtha in his 1555 translation of Pedanius Dioscorides’s book on poisons.

Another type of asphalt is called naphtha, which is a certain white and liquid form of Babylonian bitumen; given that a black form can also be found. This type [of asphalt] is so able to attract fire to itself that, even if far away, the fire will leap towards it. It is good against cataracts and against a white cloudiness that occurs in the eyes. All bitumen protects the parts of the body against inflammation, and it is good for soldering, mollifying, and resolving. It restores a mother to her proper state, during pregnancy or after [‘subida o salida a fuera’] when applied or administered as a perfume. It detects those with epilepsy [‘gota coral’], just like jet, if one uses it as perfume. Drunk with wine and beaver gland [‘castoreo’] it provokes menstruation. It is useful against persistent cough [‘tose antigua’], asthma, shortness of breath, snakebites, sciatica, and pain in one’s side. To be given in pill form, to combat lax caused by a weak stomach. Drunk with vinegar it thins coagulated blood. Dissolved with tisane and injected as an enema it cures dysentery. It is useful for coughs when used in fumigation. It mitigates the pain of aching teeth when applied as a plaster. It strengthens weakened hair when dried and applied with a surgeon’s probe [‘una tienta’]. Mixed with oat flour, wax, and nitre, and applied hot, it is useful against gout, joint pain, and lethargy.^39^

Many of the properties of petroleum listed in Laguna’s translation appear strikingly similar to those listed by Guido Mondones, whose advertising leaflet we shall examine further, and by Giuseppe Balsamo, whom was cited earlier. Echoes of Dioscorides’s description appear in Nicolás Monardes account of naphtha in the New World in his widely cited and extensively translated treatise *Historia medicinal de las cosas que se traen de nuestras Indias Occidentales*, first published in 1565.^40^ The following is from John Frampton’s 1577 translation.

There is in the Islande of *Cuba*, certaine Fountaines at the Sea side, that do cast from them a kinde of blacke Pitch, of a strong smell, which the Indians doe use in their cold infirmities. Our people doe use it there to pitche theyr Shippes, withall, for it is well neere lyke unto Tarre, and they do mingle therewith Tallowe, to make it Pitch the better. I doe beleeve that this is *Napta*, which the ancient wryters doe speake of. *Possidonto* sayeth, that there are twoo Fountaynes thereof in Babylon, one whyte and the other blacke. That which is brought from the Indias, we do use against griefes of the Mother, for that it doeth reduce the Mother in her place. And if it rise on high, then put it to the Nosethrilles, and if it come downe to the lower partes, putting thereto a wet tent with this Pitche, it causeth it to go upwarde to her place: and likewise it doeth profite, being applied to cold Infirmities, as the other Medicines do which we have spoken of. It is hotte in the second degree, and moyst in the first.^41^

The observation that ‘Indians doe use [it] in their cold infirmities’ adds a distinct element of authority that blends nicely with Western classical tradition and suggests to the reader that, between the New World and the Old, the healing properties of the substance are universally recognised. At first glance, a travelling merchant such as Mondones seems like an outlier peddling an exotic remedy that benefits from its obscure origins, but when placed in context, the complete opposite situation is revealed. David Gentilcore’s database lists eleven separate licences for selling ‘rock oil’ from the years 1560 to 1597, indicating that this medicine was not an unusual one for sale in the Italian Peninsula at the time.^42^ Petroleum was a tried and trusted remedy, and Mondones could rely on the substance’s solid reputation in both his marketing and legal defence. He also would have benefitted from the publicly accepted *pharmacopeia* at large, in which oils in general figured heavily. Additionally, legal battles over novel oils demonstrate that Mondones would have been at a relative advantage in court by selling a simple and not a concoction that invited more scrutiny and could involve recipes and multiple parties.

## Medicinal Oils in Spain: Their Medical and Legal Approval for Sale

In her chapter on ‘Contributions from the marketplace’, Michele Clouse gives a detailed account of ‘Aparicio de Zubia, a practitioner from Vizcaya (Basque region, northeast Spain) who claimed to have discovered a marvellous medicinal cure—a herbal composition especially effective for treating wounds that typically required surgical intervention’.^43^ Aparicio was a shrewd self-promoter who carefully operated within the confines of Galenic medicine while making successful practical demonstrations, including some on the battlefield. Unfortunately for him, he died before he could secure ‘… an audience with the king himself, the most coveted patron in the land’.^44^ Obtaining this prize was a task that fell to his widow, Isabel Pérez, but a dispute arose concerning the authenticity of the formula she had provided for the famous ‘Oil of Aparicio’. The legal battle was a lengthy one, no doubt in part—as Clouse notes—because the defender of the recipe was now a woman, even more liable to arouse suspicions among male medical experts. Finally, the recipe was vindicated ‘through the aid of a Dominican friar who had received Aparicio’s last confession’ and the oil’s authority as a cure lasted into the nineteenth century.^45^ In a single sentence, Juan Fragoso’s account gives us the *dramatis personae* of this early modern medical and courtroom drama.

This is the oil with the following [recipe] for which [Aparicio’s] wife gave evidence in court, by order of the members of the Council at the house of the Doctor de la Gasca to whom the case was referred. She made this declaration in the presence of Doctor San Pedro, a prosecutor for the Courts of Valladolid, and that of Diego de Burgos, apothecary to His Majesty, on the twelfth of March, in the year Fifteen Hundred and Sixty Seven.^46^

Legal experts joined forces with a medical professional to render a judgment requiring an interdisciplinary approach. On the same page as the one cited earlier, Fragoso details a similar case involving the same royal apothecary but different legal officials. The substance on trial is ‘Oil of Olmedo’, perhaps not the same as ‘Oil of Aparicio’ but still ‘not a bad substitute’.

The recipe for this oil of Olmedo is the following, for which evidence was given by order of the Council before Doctor Juan Gutierrez, *protomédico* to His Majesty, and was given in presence of the apothecary Diego de Burgos, and of Damián de Rojas, scribe, the Eighth of March in the year MDLXVI….^47^

In the first case, the matter is handled by the High Court in Valladolid, while in the second, it is the *protomédico* who intervenes, demonstrating the different paths that a drug could take on its way to final approval. Sometimes the process was drawn out and involved extensive self-promotion, as in the case for Aparicio de Zubia who ‘first had to catch the attention and win the favor of a prospective patron’ and additionally prove his medicine’s worth on the battlefield.^48^ The authorities appear to have acted more quickly in approving Olmedo’s oil, perhaps because of groundwork laid by Aparicio. This occurred even though a sample could not be procured, and consequently, the king ordered Fragoso ‘to research the oil until he uncovered it secret composition and could replicate its beneficial effects’.^49^ The *protomédico* himself was in favour of the ‘Oil of Aparicio’ if prepared properly, and he was unwittingly enshrined as an endorser of the medicine’s medical–legal authenticity. The royally approved *List for prices at which merchandise is sold* (1628) names two ‘Oils of Aparicio’, one ‘green’ (‘del verde’) and the other ‘by Fragoso’.^50^ Such oils of recent invention in Spain are akin to another ‘name brand’ concoction called ‘Oil of Matthiolo’ that does not receive the same legalistic verification and scrutiny as ‘Oil of Aparicio’ or ‘Oil of Olmedo’. The famous Italian physician and botanist Pietro Andrea Mattioli’s word was enough for early modern authorities like Fragoso, especially when Mattioli’s account included his ‘story of two thieves sentenced to death, and an experiment ordered by Pope Clement VII for testing the virtue of this oil, made by Gregorio Caravita the Bolognese surgeon, his [Mattioli’s] teacher …’.^51^ The base for the medicinal oil is the simple olive oil, but ‘simple’ in a medical sense did not necessarily mean ‘simple’ in a legal sense. Olive oil was not always itself so simple. It had potential healing properties that varied by type, and each type’s power was augmented by additional ingredients.^52^ In comparison, as we shall see further, Mondones’s ‘oli petroli’ gave him safer standing as a purveyor of medicine than somebody peddling a concoction made of oil. Still, he was likely aware that there were gaps in his legal protection and that the integrity of his substance could be called into question.

## The Case of Guido Mondones and His ‘Oli Petroli’

On 2 July 1585, a man named in court documents as ‘Guido Mondones, the Italian’ signed over power of attorney (‘carta de poder’) to the *procurador* Juan de Salazar in the city of Valladolid. The lawyer was assigned to handle a lawsuit brought against the Italian by Francisco de Gracia, an import tax collector (‘arrendador de la alcabala de peso y corrillo del aire’).^53^ Although the case involved medicine, Mondones was not accused of doing business without a licence or any similar offence. Rather, at the centre of the suit was the question of whether the oil that he had imported was taxable under Castilian law. His lawyer’s argument was a straightforward one: the oil was exempt from the ten-percent (‘diez uno’) tax because it was medicine. In order to prove this point, the exact purpose of the oil was put into evidence. The case also brings to light details of Mondones's previous legal struggles and his mastery of early modern Spanish bureaucracy, offering further insight into the survival strategies developed by Italian practitioners, academic and extra-academic alike, who made their way through Spain. The first pages of the recorded case make clear that when Mondones arrived in Valladolid in 1585, he came well-prepared with a pre-emptive legal defence in documentary form that had served him well on previous occasions. It consisted of copies of civil sentences signed in his favour and a substantial writ of royal protection (‘provisión real’) issued by the Council of the Great Office of Accounts (‘consejo de la contaduría mayor’).^54^ The earliest record in the Valladolid case states that in Madrid, on 16 May 1582,

… the Italian Guido Mondones gave an account, telling us [the council] that at great cost and effort he had brought from parts in Italy to our realms a liquid and oil that they call pretolio [*sic*], which was a thing much esteemed for curing many illnesses. And when he wished to sell it in various places in these realms, sales-tax and revenue collectors attempted to collect tax on the oil that he was selling. They caused him much trouble over this matter for which he suffered injury and damages. In order to remedy this, he asked and petitioned us for the favour of giving him our letter and writ so that nobody would request or levee the aforementioned tax on the aforementioned oil that he sells ….^55^

The writ was accompanied by a copy of ‘Law 14 of Title XVIII of the ninth book’ from the *New Compilation of the Laws of These Kingdoms* (*Nueva recopilación de las leyes destos reinos*) detailing the exceptions to the sales tax (‘alcabala’) imposed on all apothecaries. Among the many excluded medicines were ‘unguents and poultices and oils’ (‘ungüentos y emplastos y aceites’) under which category Guido Mondones’s petroleum certainly fell.^56^ Notwithstanding the weight of the central bureaucracy behind him, the Italian continually faced harassment as he made his way through Castile. His strategy for easing his difficulties was to collect ‘traslados’ or copies of favourable sentences so that he could present them as evidence when the inevitable confrontation with tax collectors sprang up. The first such conflict on record occurred in the ‘Noble city of Huete’, with approximately 1,400 residents and situated about 110 km west-southwest of Madrid. In terms of jurisdiction, it was located in the territory known as the ‘Tierra de Huete’ and today sits within the boundaries of the province of Cuenca.^57^ Documents from the second half of July 1582 outline the back and forth of arguments about whether Mondones’s medicine should be taxed under the laws for oils and distillates or free of tax as declared in the royal writ. At the time of the case, Mondones had earned ‘300 *ducados*, more or less’ from sales in the two months that he had spent in the city since his departure from Madrid with his paperwork in hand.^58^ The deputy justice (‘teniente del corregidor’) who saw the case determined that it was not proven against the defendant. If the plaintiff wished to appeal, he would have to do so at the source of Mondones’s legal defence: in front of the Council of the Great Office of Accounts in Madrid.^59^ The merchant’s troubles with tax collectors kept following him to Corral de Almaguer, a town of about 1,200 residents approximately 100 km south of Madrid, located then and now in the province of Toledo.^60^ In a copy of a document signed on 22 August 1582, Mondones testified that the local governor had granted him permission to ‘sell and publicise’ his petroleum in the town and throughout the entire district of Quintanar de la Orden. The Italian additionally demanded that his royal protection be honoured and that tax collectors ‘not bother nor perturb me, nor arrest me over this, rather that they let me go free as they have done with me in other parts’ (‘ni me molesten, ni perturben, ni detengan sobre ello sino que libremente deje [*sic*] ir según que en otras partes conmigo se ha hecho’). He also protested that each day of his arrest in the town meant a loss of ‘200 *reales* that I stop earning and lose at the market fairs’ (‘doscientos reales que dejo de ganar y pierdo en las ferias’). The mayor of the town responded favourably to Mondones’s complaint and enforced the original order, although the same official insisted that the goods on sale must be those cited in the document.^61^ The next stop on the merchant’s itinerary occurred 2 weeks later and about 40 km farther south in a town simply called ‘Alcázar’, today known as Alcázar de San Juan. Today it is part of the province of Ciudad Real, but in Mondones’s time, when its population neared 2,000, it would have had its own ‘singular jurisdiction’.^62^ In the copy of a decision dated 4 September 1582, the local magistrate showed himself as sympathetic as his colleague before him. Deferring to royal authority, the official found in favour of Mondones.^63^ At this point, the merchant must have felt relief in the short term but exasperation in the greater scheme of things. In order to avoid future lawsuits, he went back to the capital and on 26 September 1582 requested a second warrant (‘sobrecarta’), reinforcing and amplifying the previous writ and imposing a hefty fine of 100,000 maravedís against any who would defy it.^64^ This newer and harsher order from Madrid must have had the desired effect. The Valladolid court case from 1585 contains no record of any more suits until the merchant’s arrival at the city, at which point he would face two types of legal challenges: one of the sort described earlier and the other related to the quality of the medicine itself.

The first legal obstacle for Mondones was similar to those he had encountered beforehand. Within Valladolid's jurisdiction, lawsuits originating in matters of sales tax (‘alcabala’) were handled by a tribunal called the ‘Sala de los Alcaldes de los Hijosdalgo’, whose main purpose was to handle matters of noble status (‘hidalguía’). If the *hijosdalgo* ruled against the defendant, appeals were heard by the ‘Sala de Oidores’. This is precisely what happened, but not before some hardship at the hands of an import tax collector (‘arrendador de la alcabala del peso y corrillo del aire’) named Francisco de Gracia who made an appeal of his own.^65^ In court documents, Gracia testifies that, as of 9 July 1585, Mondones had been in Valladolid for 15 days, selling his petroleum and earning 200 *ducados* of which he was obligated to pay the 10 per cent tax. Gracia sought to enforce the collection by enlisting the aid of the local governor’s lieutenant (‘teniente del corregidor’), [*Juris*] Doctor Agedo de Trillo. But Mondones was quick to brandish his favourable legal sentences and writ of royal protection (‘hizo demostración de ciertas sentencias’) so that the lieutenant, like so many local officials before him, deferred to the authority of Mondones’s legal documents and declared the Italian free from paying tax.^66^ Gracia was undeterred and appealed Agedo’s judgment before the ‘Sala de los Alcaldes de los Hijosdalgo’ on 2 July, at which point Mondones granted power of attorney to his lawyer. He must have realised that he now needed a true legal professional to battle for him in court. The tax collector’s strategy was clear: he would appeal previous positive sentences on the grounds that they represented *errores in iudicando* and *errores in procedendo* (‘en grado de apelación, agravio y nulidad’) and therefore, one assumes, set no precedent for Mondones to escape taxation.^67^ The *hijosdalgo* ruled in favour of Gracia on 16 July, effectively declaring null and void the legal backing that the Italian carried with him. But the tax collector’s triumph was short-lived because Mondones’s lawyer appealed to the other *sala*, that of the *oidores* (‘judges’) on the very next day. As a consequence, the previous decision was reversed, and Mondones was victorious. Not willing to leave anything to chance, he insisted on obtaining a copy of the judge’s sentence, which was granted to him on 14 September 1585. The sentence stated that anybody acting against the judgment and attempting to collect the tax would suffer a fine of 10,000 maravedís.^68^ In this way, the question of paying taxes was settled, but it was not the end of Guido Mondones’s legal woes.

Nearly 2 weeks later, on 29 September, he faced his second and separate legal obstacle when the local governor Menén Suárez de Solís ordered that:

… Guido Modones [*sic*] not sell [his petroleum] because the governor had been informed that the aforementioned oil was not good, that it was fake and false, having been warned by many people who had bought it and found it to be of no benefit ….^69^

It seems like Mondones defied this order and confidently felt himself safe from prosecution. On the same evening that the governor issued his order, a constable in charge of vagabonds (‘alguacil de vagamundos’) was sent to the Italian’s house and confiscated two ‘*brazos* (tubes?) of liquid’ to be held in deposit, after which he put Mondones in jail.^70^ The merchant must have remained there as the governor completed his investigation, which relied upon the testimony of two physicians named in the documents as ‘Licenciado San Pedro’ and ‘Doctor Mercado’.^71^ The second figure is significant because it is Doctor Luis Mercado, Chair of Medicine at the University of Valladolid at the time, and who in seven years would be appointed royal ‘Médico de Cámara’ and replace Doctor Francisco Vallés as the nation’s top medical authority: the *Protomédico de Castilla*.^72^ These two authorities, the second very weighty indeed, would inspect the medicine and offer their conclusions. It is not clear how much time Mondones spent in custody, but he would have been relieved to hear that on 2 November 1585, the two doctors had seen:

… the aforementioned oil, examined it, performed all due diligence, and to them it appears to be the petroleum of which the authors make mention and they [the physicians] have not been able to find any signs of it being adulterated. As such, it can be used however one sees fit, as ordered by the authors, and not otherwise….^73^

On 15 November, after hearing the experts’ testimony, the ‘Sala de Oidores’ ruled in favour of Mondones, absolving him of any wrongdoing while firmly insisting that he could only continue to sell his petroleum in accordance with the uses sanctioned by the two doctors, presumedly ‘as ordered by the authors [printed medical authorities], and not otherwise’.

Given jurists’ limited knowledge on the subject, deference to medical professionals may seem like a matter of course, but this was by no means guaranteed.^74^ While we have no details of the doctors Mercado and San Pedro’s evaluation process, two other cases can give us an idea of potential obstacles that Mondones faced in the courtroom context of judging expertise. In Verona during the first two decades of the seventeenth century, there was a commission for evaluating surgeons and their procedures centered on controlling blood flow. At the same time, there were occasions that required an equally—if not more—cautious process by the same commission for approving external medicine, such as a mercury unguent, for treating the great pox or French disease, known today as syphilis.^75^ If we consider the many health claims and external uses proposed by Mondones in his advertisement, it is possible that these received the same high level of scrutiny as in the Verona cases. On other occasions, beyond scrupulousness, outright bias could stand in the way of a medical merchant’s success in court. In Valencia in 1441, during a lawsuit between an apothecary and a merchant, two physicians were called to form a commission for evaluating a substance known as ‘l’or potable’ (*auro potabili*, a drinkable gold elixir). Because the medical substance in question was of an alchemical nature, and because one of the doctors saw alchemists as obsessed with fruitless pursuits, it was possible that some of the medical expertise in this situation was biased towards suspecting the apothecary of fraudulence.^76^ If we compare this to our sixteenth-century Valladolid case, it is important to note that Mondones had elected to sell a simple (petroleum), which must have helped him avoid any alchemical controversy.^77^ In this case, the two doctors’ own biases do not appear to have interfered, and when all was said and done, they made their own deferrals to printed medical authorities. This was probably the aim of Mondones’s medico-legal strategy from the outset. He did not sell himself as an expert, but instead relied upon weighty authorities like Laguna, Brassavola and Platearius to make his claims for him. He must have known that there was a risk that his sources may have landed him in controversy or that the court would be sceptical of medical experts’ judgments, but it was only a slight gamble and certainly worth taking. The last word on the merchant to be found in the archival documents is the *sala’*s agreement, dated 27 November 1585. It promises to provide him with a copy of the sentence and warns of a fine for anybody who might attempt to contravene it.^78^ We can only imagine that, within the jurisdiction of Valladolid, Mondones was free to sell his wares and enjoyed continued success.

## Mondones’s Medical Petroleum Advertisement and the Italian Connection

Compelling evidence of doing good business in 1583 and 1584 is found in the court documents through a transcription of a handbill advertising his petroleum. As C.J.S. Thompson wrote in 1928 about London from the same time period:

The quack has always shown great skill in advertising and recognised it as the mainspring of success. Before the era of printing he had to rely solely on his lungs for haranguing his audience, but in the sixteenth century he began to appreciate the value of the press in making known his remedies and commenced to circulate hand-bills.^79^

Decades later, Roy Porter also emphasised the move towards print, describing ‘their bid for the public ear and eye through oratory, handbills, broadsides, verse, street-theatre, and, increasingly, pamphlets and newspaper publicity’.^80^ ‘Quack’ may seem like a quaint expression inapplicable to Guido Mondones, given his exoneration described above, but it is still a keyword for studying medical advertisement to this day. One also learns from more recent studies that, for England at least, extant ‘quack advertisements’ for scholarly examination are plentiful in the seventeenth century but harder to find for periods before or after.^81^ For David Gentilcore, the word is not so much ‘quack’ but ‘charlatan’ and, based on his research, handbills advertising Italian charlatans seem easier to trace across the centuries in Italy.^82^ While both England and Italy contain gaps in documenting the development of medical handbills, they offer a relative abundance of sources compared to Spain. This makes Mondones’s advertisement rather extraordinary. An extensive survey of Spanish broadsheets from 1472 to 1700 lists none printed for 1584 (the year of Mondone’s handbill), and over the entire time period, only 13 sheets printed in Salamanca (the place of the handbill) are recorded.^83^ The catalogue for the Sánchez-Quintar medical books collection at the University of Valencia lists six works printed in Salamanca in the sixteenth century, and none of these are broadsheets. Six such advertisements that are dated near the time of Mondones’s activity are from the first third of the seventeenth century.^84^ Given the context described earlier, it seems necessary to include Mondones’s advertisement in its entirety here.

Here we shall tell anew of a liquid called petroleum which by another name is called naptha as Laguna the writer of medicine tells us from page 59 until page 61, and Hieronymus Calestano, author of medicine in Spanish tells us in chapter [read ‘page’] 130 of the marvelous effects of what is called naphtha or petroleum. Many other doctors, like Antonio Musa Brassavola in his book of *Examine simplicium* and Platerius Medicus Salernitanus [corner of paper missing] —????— *De simplicibus* and many others that deal extensively with its source and origin. The aforementioned liquid comes from the mines and springs from parts of Italy. It [illegible] and is useful for all sorts of illnesses that stem from cold. Any person can drink two drams if needed and can smear himself with either a feather or a bit of cotton on any part of the body that feels cold. Afterwards, when whichever part, uncovered by any clothing, has been smeared, it can be brought near a flame and held to the heat as much as can be suffered until the liquid is consumed. Firstly, the liquid is good for gout. If the gout is caused by cold, smear where it hurts. For pain in the spleen, drink it and smear the body. For pain caused by passing stones, drink it. Also [*Item*]: for an aching belly, smear it. For aching molars and teeth, smear them or drink it to apply where it hurts. Also: for aches in the lower side, smear where it hurts. Also: for congealed nerves. Also: for persistent cough, drink it and smear the chest. Also: for worms, drink it. Also: smear the belly. Also: for aching breasts, smear them if the ache is from cold. Also: for suffocation of the mother, if the womb is fumigated from below, then by smearing with a bit of cotton and so bathe the womb with the aforementioned liquid. Then put a flame near it to fumigate underneath, and smear those parts. And if the hysteria is above, then drink a bit of it and smear the nostrils. And for epilepsy. And for snakebites, smear onto the bite because the liquid is so subtle that it will enter the flesh and consume the poison. Also: for asthma, drink it and smear the body. Also: for earache, drench a bit of cotton in it and smear it around the inner ears. Then, put these inside. Also: for sciatica, smear where one has it. For aches in the upper side, smear where one feels the pain. Also: drinking it with vinegar will dissolve blood clots. Also: for spasms. Also: for looseness that stems from weak bowels. Also: for other illnesses, as told by so many books mentioned above. Also: it mollifies and solders. And he who wishes to see it will be shown a royal writ from Madrid and from Portugal and from many other cities among the largest in Spain. The aforementioned liquid can be kept for two hundred years without it doing harm. He who has it is Guido Mondones, Italian merchant. Printed in Salamanca in the year 1584.^85^

The text for this advertisement appears suddenly in the court documents, directly after they indicate that an official has asked for a sentence to be read. But what follows is the advertising text, not any sentence. Unfortunately, this means that there is no preface for this information nor any description of the physical characteristics of the handbill, such as paper dimensions, number of pages and type of paper. Likewise, the printer is not known, though we do have a place and date. The Universal Short Title Catalogue lists no fewer than nine active printers in Salamanca in 1584, among them Lucas de Junta of the Giunti family, which had shops in Spain and Italy.^86^ While we do not know exactly where and how Mondones distributed his written advertisement or sold his actual wares, the court documents do mention that oil was packaged for sale in glass apothecary’s flasks (‘redomas’), so it is possible that he sold directly to shops.^87^ The broadsheet’s claim that he had a writ from Portugal suggests that he sought it while King Philip II (I of Portugal) resided in Lisbon between December 1580 and February 1583. This reference underscores the effort that the merchant made to secure his livelihood through the force of law.^88^ In addition to extolling the many virtues of petroleum, the advertisement matches the modern historian Gentilcore’s characterisation of how ‘The handbill was able to add to a remedy’s mystical aura for literate, partially literate, and illiterate alike’.^89^ With Mondones’s advertisement in hand, an apothecary with a substantial library would be able to check the precise page references to Andrés de Laguna’s *Pedacio Dioscorides Anazarbeo, Acerca de la materia medicinal y de los venenos mortiferos* (Antwerp: 1555, Salamanca: 1566) and Girolamo Calestani’s *Delle osservationi di Girolamo Calestani Parmigiano parte prima* (Venice: 1575). For Antonio Musa Brassavola, Mondones is likely referring to *Examen omnium simplicium medicamentorum*, and he would not have been the first person to abbreviate the title.^90^ ‘Platerius Medicus Salernitanus’ must refer to Matthaeus Platearius, author of *Circa instans*, a ‘medieval besteller’ that would later be printed at the end of the fifteenth century and many times afterwards.^91^ After checking these references, the apothecary could rest assured of the healing properties of the liquid known as ‘petroleum’ or ‘naphtha’.^92^ Despite the number of authorities he cites and the appearance of petroleum as a near cure-all, Mondones seems to have been selective in describing his remedy. For example, the merchant copies from Laguna a phrase nearly word-for-word: ‘drinking it with vinegar will dissolve blood clots’, yet the Italian omits a cure that appears in Laguna a few lines above, specifically that ‘drunk with wine and beaver gland, it provokes the menses’. On the other hand, Mondones gives more detail than Laguna about how petroleum cures snake bite.^93^ Conversely, Laguna’s petroleum-cure for gout is more elaborate and involves mixing it with barley, wax and nitre, then applying it hot; while Mondones simply indicates: ‘smear it where it hurts’. It is impossible to determine why these discrepancies exist when the merchant could have simply followed his written sources to the letter, but the differences may reflect another side of Gentilcore’s ‘mixture of orality and literacy’. Perhaps Mondones struck a balance between book-learning and empiricism, or perhaps these differences represent information gleaned from other extra-academic practitioners or conversations with apothecaries or patients.

**Fig. 1:**
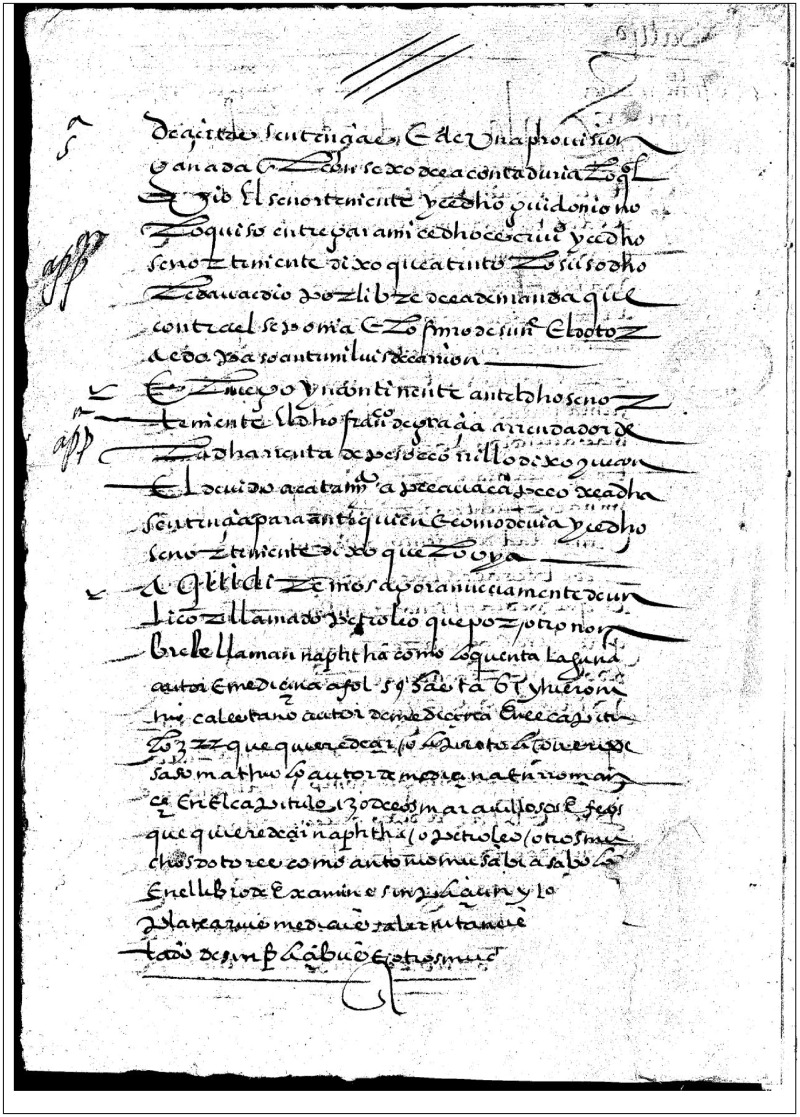
First page of handwritten copy of Mondones’s advertising handbill, starting with the line: ‘Aqui diremos…’. *Source*: Archivo de la Real Chancillería de Valladolid, Pleitos Civiles, Pérez Alonso, (F), Caja 425, 5

The documents of the legal case list Guido Mondones as a ‘merchant’, nothing more, and his advertisement is vague about the ‘mines and springs from parts of Italy’ that are the source of his petroleum. He has not dressed himself up as a medical expert, but his name could carry a certain weight and may have functioned as a pre-modern form of subliminal advertising. The court documents in the Archivo de la Real Chancillería de Valladolid are evenly divided between the spelling of ‘Mondones’ and ‘Modones’, and the former has been used in this article because it is so listed in the archive catalogue. But ‘Modones’ without the first ‘n’ is listed in the transcription of the handbill, so there is a good chance that this is how the merchant presented himself to his clientele. ‘Mondone’, without an ‘s’, survives as a rare family surname in Piedmont; but even if the merchant addressed himself in such a manner, literate consumers could still have seen a link between the name and written references to Modena and curative petroleum.^94^ If the man in question did present himself as ‘Modones’, and the other spelling was a clerical error, then the name would have implied heavily ‘from Modena’ in Spanish. Regarding the Modena-petroleum connection, Laguna writes:

In the territory of Modana [*sic*] there springs forth a certain oil called Petroleum, because it sweats out from certain rocks. It also suddenly attracts flame, and it appears very similar to white Naphtha. We may even say that it is of that kind, extremely useful for all cold illnesses of the nerves.^95^

Another of Mondones’s authoritative sources, Pietro Andrea Mattioli, updates the classical authorities’ descriptions of Naphtha and explains that while Pliny’s version of the liquid is from Astaceni in Parthia, these days, ‘…it nevertheless arises in more places, and clearly has the same effects with fire. It springs forth in Modena and other places in Lombardy, and they call it Petroleum oil or rock oil’.^96^ The family name ‘Mondones’, whether authentic or merely adopted by the merchant, was his best form of branding if we assume that Modena’s international reputation for producing medicinal petroleum preceded him.

## Conclusion

Through our modern eyes, selling petroleum may be an activity that initially throws up a figurative ‘red flag’ indicative of charlatanism, but Guido Mondones's own story inscribed in legal documents and the surrounding historical context demonstrates that he was selling a remedy that was not only quite legitimate but in fact well-reputed and quite in demand. He may not have fit neatly into the categories of either pedlar, charlatan or even ‘merchant’, but this lack of neatness is inversely proportional to the great care and pains that Mondones took to integrate himself into a system that continuously vindicated his practice, despite occasional challenges. The legitimacy of petroleum as a medicine itself was never the cause for doubt or conflict amongst the academic and legal authorities he faced, while questions of taxation and purity of the substance were. In short, Mondones was a seemingly non-hegemonic figure who was successful, in part, due to a hierarchical system and not in spite of it. Since this lack of marginalisation does not appear exceptional, the case can be added to an increasing number of examples of varied medical cultures allowing us to further explore the prominent role of accepted extra-academic practitioners on the Iberian Peninsula during the early modern period.

## Footnotes

1 John Slater, María Luz López Terrada and José Pardo-Tomás, eds, *Medical Cultures of the Early Modern Spanish Empire* (Farnham: Ashgate, 2014), 7.

2 María Luz López Terrada, ‘Las prácticas médicas extraacadémicas en la ciudad de Valencia durante los siglos XVI y XVII’, *Dynamis*, 2002, 22, 85–120.

3 Michael Stolberg, ‘Learning from the Common Folks. Academic Physicians and Medical Lay Culture in the Sixteenth Century’, *Social History of Medicine*, 2014, 27, 649–50.

4 Sabrina Minuzzi, ‘“Quick to say Quack”: Medicinal Secrets from the Household to the Apothecary’s Shop in Eighteenth-century Venice’, *Social History of Medicine*, 2019, 32, 1–33.

5 María Luz López Terrada and Carolin Schmitz, ‘Licencias sociales para sanar: La construcción como expertos de salud de curanderas y curanderos en la Castilla del Barroco’, *Studia Historica: Historia Moderna*, 2018, 40, 158.

6 David Gentilcore, *Medical Charlatanism in Early Modern Italy* (Oxford: Oxford University Press, 2006). M. A. Katritzky, *Women, Medicine and Theatre, 1550**–**1750: Literary Mountebanks and Performing Quacks* (Aldershot: Ashgate, 2007), 153–54.

7 The *gabacho* (a derogatory term for Frenchman) pedlar was the typical staple, but even *gabachos* could mix some Italian into their comical jargon onstage, a reminder that Italian-ness could be equally strong as a marker of foreignness.

8 Some historians have given up on telling these stories, while others thankfully have not. ‘The medical ideas of the lower classes, consisting largely of “out-of-date” or “misunderstood elite medicine,” were part of the medical penumbra. For a culture that was based on oral tradition and collective memory, the problem for the historian is always going to be one of access. On the basis of the only sources available, “saturated in the prejudices and assumptions of the social elite,” there is no point in attempting to “inventorize, let alone geneologize or describe” the wide range of beliefs about healing Laurence Brockliss and Colin Jones, *The Medical World of Early Modern France* (Oxford: Clarendon Press, 1997, 274)’. David Gentilcore, ‘Was There a "Popular Medicine" in Early Modern Europe?’, *Folklore*, 2004, 115, 158. For a felicitous counterexample, see how medical knowledge was transmitted to an understanding doctor who wrote it down in Michael Stolberg, cited earlier. For examples of stories of extra-academics (apothecaries specifically) told through the media of legal proceedings, see the article by Carmel Ferragud and José Ramón Bertomeu Sánchez and book by Pastor Frechoso, both cited separately further.

9 Gentilcore, *Medical Charlatanism in Early Modern Italy*, 121. Robert Jütte also reminds us that we are in a period of ‘proto-professionalization’ and that ‘In medieval and early modern times the boundary between “folk medicine” and university-taught medicine was still diffuse and vague despite the vehement delimitation efforts of the medical fraternity.’ For Jütte, ‘folk medicine’ is synonymous with a specific sort of ‘lay medicine’, in which diagnosis and treatment starts within the patient’s immediate social circle, which then looks for ‘therapeutic alternatives’. Robert Jütte, ‘Medical Pluralism in Early-Modern Germany’, in Robert Jütte, ed., *Medical Pluralism: Past-Present-Future* (Stuttgart: Steiner Verlag, 2013), 26–28. Jütte’s edited collection also contains essays on France and Italy, for comparison. David Gentilcore, ‘Medical Pluralism and the Medical Marketplace’, in Jütte, *Medical Pluralism*, 45–55. Matthew Ramsey, ‘“Medical Pluralism”in Early Modern France’, in Jütte, *Medical Pluralism*, 57–80.

10 María Luz López Terrada and Àlvar Martínez Vidal, ‘El Tribunal del Real Protomedicato en la Monarquía hispánica’, *Dynamis*, 1996, 16, 17–19, 17 (my translation).

11 A follow-up issue to the article cited earlier illustrates how, in only 6 years, modern historians’ views on the subject evolved, granting more agency and variety to practitioners, operating outside of institutional authority. See Rosa Ballester, María Luz López Terrada and Àlvar Martínez Vidal, ‘La realidad de la práctica médica: el pluralismo asistencial en la monarquía hispánica (ss. XVI- XVIII)’, *Dynamis*, 2002, 22, 21–28, 22.

12 Enrique Jorge Enríquez, *Retrato del perfecto médico* (Salamanca: Juan y Andres Renaut, 1595), 60–61.

13
*Ibid*.,110.

14 María Soledad Campos Díez, *El Real Tribunal del protomedicato castellano, siglos XIV**–**XIX* (Cuenca: Ediciones de la Universidad de Castilla-La Mancha, 1999), 321.

15
*Ibid*., 53.

16
*Ibid.*, 54.

17
*Ibid*., 64.

18
*Ibid*., 62.

19
*Actas de las Cortes de Castilla: Contiene las de Madrid, celebradas el año 1563*, 2 vols (Madrid: Imprenta nacional, 1861–62), I, 104.

20 Consider the title of William Eamon, ‘The Charlatan's Trial: An Italian Surgeon in the Court of King Philip II, 1576–77’, *Cronos*, 2005, 8, 3–30.

21 Francisco Díaz, *Tratado nuevamente impreso, de todas las enfermedades de los Riñones, Vejiga, y Carnosidades de la Verga, y Urina*, etc. (Madrid: Francisco Sánchez, 1588), 322v.

22 Juan de Mal Lara, *La philosophia vulgar: primera parte que contiene mil refranes glosados* (Sevilla: Hernando Diaz, 1568), 283r-v.

23 Gentilcore, *Medical Charlatanism in Early Modern Italy*, 295–96.

24 Sebastián de Covarrubias Orozco, *Tesoro de la lengua castellana o española* (Madrid: Luis Sánchez, 1611), 291v-92r.

25 Cristóbal Suárez de Figueroa, *Plaza universal de todas ciencias y artes*: *parte traducida de toscano y parte compuesta por el doctor Christoval Suarez de Figueroa* (Madrid: Luis Sánchez, 1615), 325r.

26
*Ibid.*

27 José Rodríguez Guerrero, ‘La Primera Gran Red Comercial de un Medicamento chymico Vittorio Algarotti y su Quintaesencia del Oro Medicinal’, *Azogue*, 2008–2009, 6, 12–67, 39–41.

28
*Ibid*., 46.

29 For Algarotti’s qualifications, see *Ibid*., 12

30 Gentilcore, *Medical Charlatanism in Early Modern Italy*, 360. Rodríguez Guerrero explains how Quintilio, ‘…since he was no physician, nor familiar with medical theories, was forced to copy the arguments of his predecessor’. Rodríguez Guerrero, ‘La Primera Gran Red Comercial’, 47–48. But since Quintilio did not hide the fact that his copies were translated from Italian, it would be difficult to condemn him—then or now—for an extreme transgression like a ‘cat’s cradle of forgery and plagiarism, imitation and translation’. Nick Groom, ‘Forgery, Plagiarism, Imitation, Pegleggery’, in Paulina Kewes, ed., *Plagiarism in Early Modern England* (New York: Palgrave Macmillan, 2003), 83.

31 Alessandro Quintilio, *Relación y memoria de los maravillosos efectos y notables provechos que han hecho y hacen los polvos blancos solutivos de la quinta esencia del oro que compone y hace Alexandro Quintilio* (Madrid: Luis Sánchez, 1616), ‘relación sumaria’ at the front of book.

32
*Ibid*.

33 I have not yet been able to consult the document, but it is described this way in the SAVEX catalogue (<http://savex.cult.gva.es/flora/jsp/index_opac.jsp> accessed on 2 April 2019) in the Archivo del Reino de Valencia. It is catalogued as: Archivo del Reino de Valencia, Manaments y Empares, a. 1609, Libro 11, mano 108, fol. 1r-3r.

34 María Luz López Terrada, ‘El control de las prácticas médicas en la Monarquía Hispánica durante los siglos XVI y XVII: el caso de la Valencia foral’, *Cuadernos de historia de España*, 2007, 81, 91–113, 106–07. In Gentilcore’s database of charlatans, the only cases of medicine labelled as ‘germanica’ or ‘germania’ are from the eighteenth century and unlikely to provide any clues as to the contents of Jusepe Balsamo’s creation.

35 Another useful point of comparison would be the case of Giuseppe Felice Maria Scutellio (1666–1730), described in a study by Sabrina Minuzzi. ‘From the mid-sixteenth century onwards, alongside the “professional” quacks who mostly sold secrets in the streets and squares by resorting to theatrical gimmicks, there were others who applied for licences to produce and sell secrets that had been devised thanks to their daily ‘diligent study and investigation.’ Giuseppe Scutellio was one of these many ‘“particular people” […]’. Minuzzi, ‘“Quick to say Quack”’, 2. We can safely say that Guido Mondones was not one of these ‘particular people’, not dedicated to secrets nor establishing a reputation based on these, nor seeking licenses for them; but nor was he merely a ‘gimmicky quack’. Instead, he found other ways, explained further, to use the legal system and medical authorities to build and maintain his reputation as a respectable medical merchant. For a comprehensive look at purveyors of secrets in an early modern Venetian context and for good examples of the intersection between licensing, legality and medicine, see Sabrina Minuzzi, *Sul filo dei segreti: Farmacopea, libri e pratiche terapeutiche a Venezia in età moderna* (Milan: Unicopli, 2016).

36 ‘Following the recommendations of Avicenna, petroleum came to be recommended by many of the leading physicians and surgeons of the western Middle Ages.’ Grantley McDonald, ‘Georgius Agricola and the Invention of Petroleum’, *Bibliothèque d'Humanisme et Renaissance*, 2011, 73, 354–55.

37 R. J. Forbes, ‘Advertizing the Medicinal Properties of Petroleum’, in *Studies in Early Petroleum History* by R. J. Forbes (Leiden: Brill, 1958), 98–114.

38
*Ibid*., 116, 118, 120. Also, ‘So far only one type of such a pamphlet has turned up which is devoted to the St. Quirinus oil of Tegernsee, which source of supply must have found the Italians active and dangerous competitors’. *Ibid*., 109. ‘Ancient medical authorities’ are Forbes’s words. *Ibid*., 110.

39 Andrés de Laguna, *Pedacio Dioscorides Anazarbeo, Acerca de la materia medicinal y de los venenos mortíferos* (Salamanca: Mathías Gast, 1566), 59.

40 Ralph Bauer, ‘The Blood of the Dragon: Alchemy and Natural History in Nicolás Monardes’s *Historia medicinal*’, in Slater, López-Terrada and Pardo-Tomás, *Medical Cultures of the Early Modern Spanish Empire*, 67–90, 67.

41 Nicolás Monardes, *Joyfull newes out of the newe founde worlde*, John Frampton (trans), (London: William Norton, 1577), 6r–6v. Monardes writes of Cuba, but petroleum deposits were discovered in the New World beforehand. ‘In a letter from Queen [Regent] Isabella the Catholic directed to Royal Officials of the city of Nueva Cádiz, dated the 3rd of September 1536, one finds written: “Some people have brought to these Kingdoms a petroleum oil [“azeite petrolio”] from a spring on said island [Cubagua] and because here it has appeared to be of value, I command that all ships sailing from that island send me as much as possible.” The order of the Sovereign leaves it quite clear that already by the 3rd of September the aforementioned petroleum had arrived in Spain’. Joaquín A. Ramírez, *Petróleo, energía y opinión pública* (Madrid: Joaquín A. Ramírez, 1956), 54.

42
*Ibid.*

43 Michele L. Clouse, *Medicine, Government and Public Health in Philip II's Spain: Shared Interests, Competing Authorities* (Farnham, Surrey: Ashgate Publishing, 2011).

44
*Ibid*., 79.

45
*Ibid.*, 81.

46 Juan Fragoso, *Cirugía universal, ahora nuevamente enmendada, y añadida en esta quinta impresión* (Alcalá: Juan García, 1592), fol. 251r. ‘Doctor de la Gasca’ must refer to Diego de la Gasca, a doctor in laws and canon who ‘began his public career as an *oidor* [judge] of the Chancilleria [a sort of high court] in Valladolid and in March of 1552 was appointed member of the Royal Council of Castile, the most important administrative body that existed in the Spanish monarchy under the Hapsburgs’. Teodoro Hampe Martínez, ‘Don Pedro de la Gasca y la proyección del mundo universitario salmantino en el siglo XVI’, *Mélanges de la Casa de Velázquez*, 1986, 22, 171–96, 192.

47
*Ibid.*

48 Clouse, *Medicine, Government and Public Health*, 78.

49
*Ibid.*, 86.

50 Juan González, *Tasa de los precios a que se han de vender las mercaderías y otras cosas de que no se hizo mención en la primera Tasa* (Madrid: Juan González, 1628), fol. 48v.

51 Fragoso, *Cirugía universal*, fol. 252r.

52 And see, for example, Luis de Oviedo’s explanation of how to prepare oil of rue: ‘One must understand that when making oils from olive oil, cold oils must be made with *omphancino* oil, which is oil from green unripe olives, and not ripe ones; and hot oils must be made with sweet, mature oil. I take this, by the letter, from Galen ….’ Luis de Oviedo, *Método de la colección y reposición de las medicinas simples* (Madrid: Luis Sánchez, 1595), fol. 347r.

53 Archivo de la Real Chancillería de Valladolid, Pleitos Civiles, Pérez Alonso, (F), Caja 425, 5, fols. 17r–18v. The full title of Francisco de Gracia’s office is found on fol. 26r.

54
*Ibid.*, fols. 1r–1v.

55 ‘…Guido Mondones italiano nos hizo relación diciendo que con mucha costa y trabajo había traído de las partes de Italia a estos nuestros reinos un licor y aceite que llaman pretolio [*sic*] que era cosa muy estimado para muchas enfermedades, y que queriéndole vender en algunos lugares de estos reinos los arrendadores de las alcabalas y rentas de ellos les pretendían llevar alcabala del aceite que vendiese, y sobre ello le hacían muchas molestias de que recibía agravio y daño, y para remedio de ello nos suplicó y pidió por merced le mandásemos dar nuestra carta y provisión para que del dicho aceite que así vendiese no se le pidiese ni llevase la dicha alcabala…’. *Ibid*., fols. 4r–4v. I include this text in the original language for scholars who may not otherwise have access to the document. Here and in following citations, I have modernised the spelling, accentuation and capitalisation. I have also added punctuation for ease of reading.

56 The most up-to-date version of this law would have been found in *Cuaderno de lo que se añadió al libro de la nueva Recopilación en esta segunda impresión que se imprimió este año de MDLXXXI* (*Booklet of what was added to the book for the New Compilation in its second printing that was printed in the year MDLXXXI*) (Alcalá: Juan Íñiguez de Lequerica, 1581), fol. 16r.

57 It is important to note that, by the time of Mondones’s arrival in the Peninsula, the Spanish Crown’s financial system had undergone a process of decentralisation, providing much power (and potential for abuse) to the local authorities in the collection of the *alcabala*. This may, in part, explain the merchant’s constant need to prove himself despite carrying documents from the capital. Pilar Zabala Aguirre, *Las alcabalas y la Hacienda Real en Castilla: Siglo XVI* (Santander: Universidad de Cantabria, 2000), 309. I approximate the population of Huete at the time by using figures from 1561 (1,457) and 1591 (1,340). Julián Montemayor, ‘La red urbana en Castilla la Nueva en los siglos. XVI y XVII’, *Brocar*, 1987, 13, 143.

58 Archivo de la Real Chancillería de Valladolid, Pleitos Civiles, Pérez Alonso, (F), Caja 425, 5, fol. 9r. ‘An *escudo* coin [equal to a *ducado*] in the 1580s was worth 400 *maravedís.* A *real* coin was “usually worth 34 *maravedís*”.’ Mary Elizabeth Perry, *Crime and Society in Early Modern Seville* (Hanover, New Hampshire: University Press of New England, 1980), 265. Thus, 300 *ducados* equalled 120,000 maravedís, far exceeding the yearly salary of professors of medicine at the Universities of Álacala and Salamanca, which averaged between 40,000 and 80,000 maravedís. Clouse, *Medicine, Government and Public Health*, 58n. The average retail price of medicinal petroleum was 64 *reales* per pound according to figures from Valladolid over the period from the mid-sixteenth to late seventeenth centuries. A total of 300 *ducados* of profit could therefore be estimated to equal 55 pounds of petroleum, but if this seems like too much volume, then perhaps Mondones was selling at much higher rates. For comparison, an apothecary’s sweet almond oil averaged 7.35 *reales* per pound or roughly one-ninth the price of petroleum. Linseed oil averaged 1.62 *reales* per pound or roughly one-fortieth of the cost. Felix Francisco Pastor Frechoso, *Boticas, boticarios y materia médica en Valladolid: Siglos XVI y XVII* (Valladolid: Junta de Castilla y León, 1993). For further comparison, in 1580, a pound of olive oil cost 16 maravedís, meaning that Mondones’s petroleum sold at roughly 140 times as much as this food staple. Cristóbal Espejo, ‘La carestía de la vida en el siglo XVI y medios de abaratarla’, *Revista de Archivos, Bibliotecas y Museos*, 1920, 41, 36–54, 169–224, 329–54; 342.

59
*Ibid*., fol. 11r.

60 Montemayor, ‘La red urbana en Castilla la Nueva’, 143.

61
*Ibid*., fols. 12v–13v.

62
*Ibid*., 143. Ignacio Javier Ezquerra Revilla, ‘Singularidad jurisdiccional del Priorato de San Juan en La Mancha en tiempo del Quijote: la comisión de la langosta del licenciado Salvatierra (1619)’, in Francisco Ruiz Gómez and Jesús Molero García, eds, *La orden de San Juan en tiempos del Quijote* (Ciudad Real: Universidad de Castilla-La Mancha, 2010), 359–78.

63
*Ibid.*, fol. 14r.

64
*Ibid.*, fol. 8r. The amount of the fine and the wording of this particular document closely matches that of a *pragmática* (‘decree’) aimed at protecting merchants in Seville in 1540. Vicente de Cadenas y Vicent, *Carlos I de Castilla, señor de las Indias* (Madrid: Hidalguía, 1988), 258. It is also 10 times the amount of the penalty first issued by the Royal Council against those who would attempt to collect tax from Mondones. Archivo de la Real Chancillería de Valladolid, Pleitos Civiles, Pérez Alonso, (F), Caja 425, 5, fol. 4v.

65 Archivo de la Real Chancillería de Valladolid, Registro de Ejecutorias, Caja 1538, 17, fol. 1r. For more information on the *sala* and the appeals process, see Vicente de Cadenas y Vicent, *Pleitos de Hidalguía que se conservan en el Archivo de la Real Chancillería de Valladolid, extractos de sus expedientes: siglo XIX. Tomo I, Letra A* (Madrid: Hidalguía, 1976), 32–33.

66 Archivo de la Real Chancillería de Valladolid, Registro de Ejecutorias, Caja 1538, 17, fols. 1r–1v.

67 Archivo de la Real Chancillería de Valladolid, Pleitos Civiles, Pérez Alonso, (F), Caja 425, 5, fol. 18r. I base my translation of legal terms on information provided in Enrique Manuel Falcón, *Código procesal civil y comercial de la nación: anotado, concordado, comentado*, vol. 2 (Buenos Aires: Abeledo-Perrot, 1982), 362.

68 Archivo de la Real Chancillería de Valladolid, Registro de Ejecutorias, Caja 1538, 17, fol. 2v.

69 ‘…Guido Modones no lo vendiese porque era informadao que el dicho aceite no era bueno, y era fingido y falso, por haber tenido avisos de muchas personas que lo habían comprado y no les había hecho provecho…’. Archivo de la Real Chancillería de Valladolid, Registro de Ejecutorias, Caja 1541, 61, fol. 1r.

70
*Ibid.*, fol. 1v.

71
*Ibid.*, fol. 2r.

72 Campos Díez, *El Real Tribunal del protomedicato*, 378.

73 ‘…el dicho aceite y examinádolo, y hecho las diligencias nescesarias, y les parece ser el pretolio [sic] de de que los autores hacen mención y no han podido hallar en ello hallar [sic] señas de estar adulterado, y que por tal se puede gastar como mire quien lo usare. Que les [sic] use para que los autores lo mandan y no más…’. *Ibid.*, fol. 2v.

74 For example, consider the scepticism of seventeenth-century jurists Giovanni Battista De Luca and Antonio Maria Cospi towards medical expertise. See Silvia de Renzi, ‘Witnesses of the Body: Medico-legal Cases in Seventeenth-century Rome’, *Studies in History and and Philosophy of Science*, 2002, 33, 225 and Silvia de Renzi, ‘Medical Expertise, Bodies, and the Law in Early Modern Courts’, *Isis*, 2007, 98, 320, respectively.

75 Alessandro Pastore, *Il medico in tribunale: la perizia medica nella procedura penale d'antico regime (secoli XVI-XVIII)* (Bellinzona: Casagrande, 1998), 188–89.

76 Carmel Ferragud and José Ramón Bertomeu Sánchez, ‘Mirades sobre l'alquímia a la Valencia baixmedieval: Un procés judicial sobre la producció de l'or potable (1440–1445)’, *Afers*, 2015, 82, 437.

77 See the example of Marco Cornacchini in relation to that of Quintilio, in which Cornacchini was embroiled in a controversy with his peers over his antimony powders. Mar Rey Bueno, ‘*Prolongatio vitae*: prácticas alquímicas, remedios secretos y promesas de salud en la España Moderna’, *Azogue*, 2010–2013, 7, 395–96.

78
*Ibid.*, fol. 3r.

79 C. J. S. Thompson, *The Quacks of Old London* (New York: Brentano’s, 1928), 27.

80 Roy Porter, ‘The Language of Quackery in England, 1660–1800’, in Peter Burke and Roy Porter, eds, *The Social History of Language* (Cambridge: Cambridge University Press, 1987), 78.

81 ‘…The British Library boasts a magnificent collection of metropolitan quack handbills dating from the late seventeenth century but no archive, so far as I am aware, possesses the equivalent bills for, say a century later.’ Roy Porter, *Quacks: Fakers and Charlatans in English Medicine* (Charleston, South Carolina: Tempus, 2000), 13. In a recent study by Roberta Mullini, she relies on three British Library collections covering 1675–1715, 1660–1716 and 1528–1715 but explains that this last one ‘…will be seldom taken into account, since it mainly collects sundry items, i.e. not medical adverts published by irregular practitioners.’ Roberta Mullini, *Healing Words: The Printed Handbills of Early Modern London Quacks* (Frankfurt am Main: Peter Lang, 2015), 20.

82 Gentilcore, *Medical Charlatanism in Early Modern Italy*, 340–58.

83 Alexander S. Wilkinson, Alejandra Ulla Lorenzo and Alba de la Cruz, ‘A Survey of Spanish Broadsheets: 1472–1700’ in Andrew Pettegreee, ed, *Broadsheets: Single-sheet Publishing in the First Age of Print* (Leiden: Brill, 2017), 61.

84 Juan Antonio Micó Navarro, *Catálogo del fondo Sánchez-Quintanar* (Valencia: Universitat de València-C.S.I.C, 1994), 6. The catalogue entries for documents that I have identified as datable handbills from the cited time period are found on pages 43, 50, 135, 194, 217 and 234. The first document, that of ‘Giuseppe Balsamo’, contains no date but can be roughly dated based on other information, referred to earlier in this article.

85 Archivo de la Real Chancillería de Valladolid, Pleitos Civiles, Pérez Alonso, (F), Caja 425, 5, fols. 1v-2v. See Figure 1 for an image of the first page. Also see the ‘Appendix’ for a full transcription, with modernised spelling of the handbill.

86 Angela Nuovo, *The Book Trade in the Italian Renaissance* (Leiden: Brill, 2013), 55.

87 Archivo de la Real Chancillería de Valladolid, Pleitos Civiles, Pérez Alonso, (F), Caja 425, 5, fol. 10r. After citing the purchase of 200 ‘redomas’ in 1579 in Burgos, at 34 *maravedís* a piece, Alberto C. Ibáñez Pérez explains that ‘Raw materials for making medicines were purchased from merchants by the apothecaries, except for an important portion of these, such as herbs and some plants, which the apothecary cultivated and gathered by hand from a small garden…’. Alberto C. Ibáñez Pérez, *Burgos y los burgaleses en el siglo XVI* (Burgos: Ayuntamiento de Burgos, 1990), 526.

88 Geoffrey Woodward, *Philip II* (New York: Routledge, 1992), 81.

89 Gentilcore, *Medical Charlatanism*, 314.

90 Juan Lorenzo Palmireno cites the doctor thusly when writing about a legendary emerald dish and its relationship to the Holy Grail: ‘Musa Brasavolo en su libro de *Examine simplicium* le llama *Paropsydes Domini*.’ Juan Lorenzo Palmireno, *Vocabulario del Humanista* (Valencia: Petri à Huete, 1569), 75.

91 Iolanda Ventura, 'Un best-seller farmaceutico medioevale tra produzione di libri e pratiche di lettura: il Circa instans attribuito a Platearius’, in N. Bouloux, A. Dan and G. Tolias, eds, *Orbis disciplinae: Hommages en l’honneur de Patrick Gautier Dalché* (Turnhout: Brepols, 2017), 628–30.

92 The Brasavola and Platerius references in the document are less precise because of paper damage and may refer to any number of books. Likely candidates for Brasavola are either *Examen omnium simplicium medicamentorum, quorum in officinis usus est* or *Examen omnium simplicium quorum usus in publicis*, which together went through 10 printings from 1536 to 1556. *De simplicibus* by ‘Platearius Medicus Salernitanus’ refers to either a manuscript of *Tractatus de simplicibus medicinis*, also known as *Circa instans*, from the medieval Salerno school of medicine or a printed version thereof.

93 See citation above from, de Laguna, *Pedacio Dioscorides Anazarbeo*, 59.

94 I am grateful to Sabrina Minuzzi for this information about the Mondone family name in Piedmont.

95 de Laguna, *Pedacio Dioscorides Anazarbeo*, 60–61.

96 Girolamo Calestani, *Delle osservationi di Girolamo Calestani Parmigiano parte prima* (Venice: Vincenzo Valgrisi, 1575), 130.

## Notes

Dr Ted L.L. Bergman is a Lecturer in Spanish at the University of St Andrews. He has published widely on early modern Spanish theatre, mainly focusing on humour or criminality and is currently working on a book about lawbreaking, peacekeeping and theatricality. Dr Bergman’s latest research area is popular medicine, charlatans and performance in Early Modern Spain, and he was awarded a Wellcome Trust Medical Humanities Small Grant to pursue this research.

## Appendix

[Transcribed and modernised text from court documents that themselves transcribe Guido Mondones’s advertising handbill]

Aquí diremos ahora nuevamente de un licor llamado petróleo que por otro nombre llaman naphtha, como lo cuenta Laguna, autor de medicina a fol. 59 hasta 61 y Hieronymus Calestano, autor de medicina en romance, en el capítulo 130 de los ‘maravillosos efectos’. Que quiere decir naphthas o petróleo otros muchos doctores en el libro de *Examine sinpiciun* [*sic*] y Platerius Medicus Salernitanus… [corner of paper missing] … tado de *Simplicibus* y otrosí muchos … [corner of paper missing] … que tratan largamente el fundamento del cual viene de las minas y fuentes de las partes de Italia el dicho licor. Presta [???] y sirve para todo género de enfermedades que proceden de frialdad. Cualquier persona puede beber dos adarmes si tuviere necesidad e puede untarse con una pluma o con un poco de algodón en cualquier parte que sintiere frialdad y, después de untada la parte que fuere, llegarla a la lumbre e tenerla al calor que pudiere sufrir hasta que el licor se consuma sin ropa encima. Primeramente, la gota si es de frialdad, es untarse donde la tuviere. Y dolor de bazo: beberlo y untarse la persona. Y para dolor de piedra: beberlo. Ítem para dolor de vientre: untarlo. Ítem para las muelas y dientes: untarlos o beber de ponerlo a la parte donde doliere. Ítem para dolor de ijada: untar donde es dolor. Ítem para nervios cuajados. Ítem para tos vieja: beberlo y untar los pechos. Ítem para lombrices: beberlo. Ítem: untar la barriga. Ítem para dolor de pecho: untarlos si fuere de frío. Ítem para mal de madre de mujeres: si la madre se ahuma abajo, untar un poco de algodón y bañarle con el dicho licor, y llegarle una vela encendida de con ella sahumarse por abajo y untar aquellas partes; y si estuviere arriba: beber un poco de ello y untarse las narices. Y para gota coral y para mordeduras de serpientes: untar encima de la mordedura porque es tan sutil que entra dentro la carne y consume la ponzoña. Ítem para asma: beber y untar su persona. Ítem para dolor de oídos: bañar un poco de algodón y untar alrededor de los oídos, y después meterlos dentro. Ítem para Ciática: untar donde la tuviere. Para dolor de costado: untar donde fuere el dolor. Ítem bebiéndolo con vinagre deshace los cuajarones de sangre. Ítem para calambre. Ítem para cámaras que proceden de franquezas. Ítem para otras enfermedades como lo cuentan tantos libros arriba dichos. Ítem morifica [*sic*] y suelda. Y [a] quien lo quisiere ver l mostrará un provisión de Madrid y de Portugal, y de otras muchas ciudades de las principales de las Españas. El dicho licor se puede guardar doscientos años sin que se dañe el que lo tiene es Guido Modones, italiano mercader. Impreso en Salamanca, año 1584.^97^

